# Gout—Cold-Water Treatment—Translation to the Heart and Stomach—Death

**Published:** 1844-02-10

**Authors:** 


GOUT—COLD-WATER TREATMENT—TRANSLATION TO THE HEART AND STOMACH—DEATH.

Mr. Baker, a surgeon at Uxbridge, was attacked with regular gout in the feet, which continued nearly stationary for three days, unattended with fever, or any material inconvenience. The fourth night was restless, the pain and inflammation increasing, but still confined to the feet. Mr. Baker resolved to try the treatment recommended by Dr. Kinglake, and accordingly applied, first, cold air, then sponging with cold water, and, lastly, cloths dipped in iced water, with manifest relief. About five hours afterwards the pain was quite gone, and the pulse 78. On getting out of bed, however, a fainting fit came on, which was removed, and he took some breakfast. A slight inflammation was observed in the knees, which was removed by the ice water. Four hours afterwards he was found by Mr. Edlin, who had been called into him, lying on his back, with difficult hurried respiration, cold extremities, and quick fluttering and intermittent pulse. He complained of palpitation of the heart and icy coldness in the stomach; he had vomited several times, and a cold sweat had broken out on the skin. Some warm brandy and water was immediately given, and followed by a draught, containing æther, opiate confection, and camphor julep; bladders of hot water were applied to the region of the stomach, the knees, the hands, and the feet, in the hope of inducing a return of the disease to the extremities, and the draught was repeated, with the addition of some musk. The pain and oppression of the breathing now began to decline, and a gentle perspiration broke out on the skin, which continued the remainder of the afternoon. In the evening, by the advice of Dr. Haygarth, the extremities were fomented; some sleep was obtained, and for two days no great change of symptoms occurred; the following day, however, another shivering fit and attack of gout in the stomach took place, and, after lingering three days longer, the patient died.

GOUT—COLD-WATER TREATMENT—FREQUENT RELAPSE—TRANSLATION TO THE HEAD AND STOMACH—ULTIMATE RECOVERY.

